# Vimentin Intermediate Filaments Mediate Cell Morphology on Viscoelastic Substrates

**DOI:** 10.1021/acsabm.1c01046

**Published:** 2022-01-07

**Authors:** Maxx Swoger, Sarthak Gupta, Elisabeth E. Charrier, Michael Bates, Heidi Hehnly, Alison E. Patteson

**Affiliations:** † Physics Department, Syracuse University, Syracuse, New York 13244, United States; ‡ BioInspired Institute, Syracuse University, Syracuse, New York 13244, United States; ¶ Institute of Medicine and Engineering, University of Pennsylvania, Philadelphia, Pennsylvania 13210, United States; § Biology Department, Syracuse University, Syracuse, New York 13244, United States

**Keywords:** cells, biomaterials, cell spreading, cytoskeleton, vimentin, cell adhesion, hydrogels

## Abstract

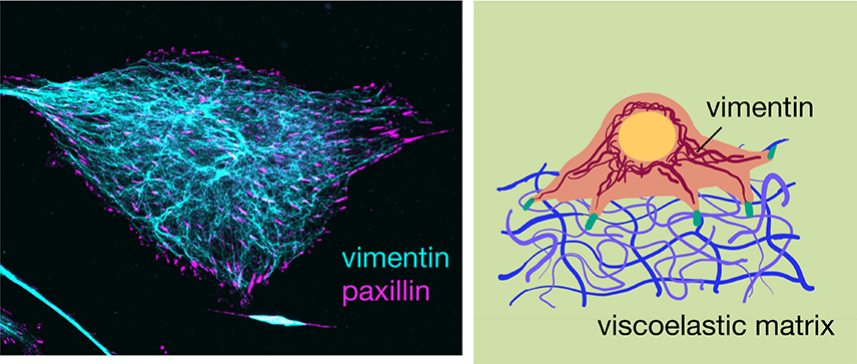

The ability of cells to take and change shape is a fundamental feature underlying development, wound repair, and tissue maintenance. Central to this process is physical and signaling interactions between the three cytoskeletal polymeric networks: F-actin, microtubules, and intermediate filaments (IFs). Vimentin is an IF protein that is essential to the mechanical resilience of cells and regulates cross-talk among the cytoskeleton, but its role in how cells sense and respond to the surrounding extracellular matrix is largely unclear. To investigate vimentin’s role in substrate sensing, we designed polyacrylamide hydrogels that mimic the elastic and viscoelastic nature of *in vivo* tissues. Using wild-type and vimentin-null mouse embryonic fibroblasts, we show that vimentin enhances cell spreading on viscoelastic substrates, even though it has little effect in the limit of purely elastic substrates. Our results provide compelling evidence that vimentin modulates how cells sense and respond to their environment and thus plays a key role in cell mechanosensing.

## Introduction

Living cells are exquisite sensors of their environment. Cells translate extracellular chemical stimuli into finely tuned signals that alter cell structure, function, and gene expression. Cells do not solely respond to their environment by means of chemical sensing but also through physical-sensing mechanisms.^1^ Mammalian cells sense surfaces as a consequence of cellular adhesions with the extracellular matrix and the cell’s actomyosin machinery, which generates cellular forces.^2−5^ Cells grown on stiff substrates assemble actin stress fibers,^6^ increase spread area,^7,8^ upregulate expression of cytoskeletal proteins,^8^ and increase cell adhesion and contractility.^9,10^ There are now several identified key molecules and signaling pathways that give rise to cell surface sensing, such as talin,^11^ focal adhesion kinases,^12^ and YAP/TAZ.^13^ Despite this molecular knowledge, we still do not yet fully understand how cells “feel” their environment.

One major challenge in cellular mechanosensing studies is defining the forces cells feel. These forces depend on the mechanical properties of the cell environment but also on cellular deformability. Both the cell and its tissue microenvironment exhibit nonlinear viscoelastic mechanical properties, capable of dissipating applied stresses on time scales relevant to cellular mechanical sensing.^14−16^ On viscoelastic substrates, stresses imposed by a spreading cell dissipate and cell spreading is set by a balance between the stress relaxation time scales of the viscoelastic substrate and the cell focal adhesion turnover rate.^17^ Thus, the cell’s sense of touch depends on the forces it can generate and the rates at which it can probe its environment.

Central to the ability of cells to move and generate stress is the cytoskeleton.^18^ The cell cytoskeleton is composed of three polymeric networks: F-actin, microtubules, and intermediate filaments (IFs). The shear modulus of the cytoskeleton is thought to be dominated by F-actin and microtubules. Networks formed by intermediate filaments are softest among the cytoskeletal networks; however, they are capable of significantly more strain-stiffening behavior^19,20^ and are more resistant to breakage.^21,22^ New studies are highlighting the importance of intermediate filaments, such as vimentin, in providing cells with viscoelasticity and the mechanical strength to withstand large and repeated stresses without damage.^23−25^ Currently, the role of vimentin in substrate sensing is largely unclear. The loss of vimentin can have opposite effects on cell spreading depending on whether the underlying elastic substrate is soft or stiff: on soft substrates, the loss of vimentin decreases cell spread area, whereas on stiff substrates, it increases the cell spread area.^23,26^ In the range of physiological-relevant stiffnesses, on the order of 1–10 kPa in shear moduli, the spread areas of wild-type and vimentin-null mEF, however, are nearly indistinguishable.^23^ The role of vimentin in the mechanical resilience of cells^23,27,28^ and its role in focal adhesion assembly,^29,30^ however, suggest that, despite the modest effect of vimentin on elastic surfaces, their effects in more physiologically relevant viscoelastic settings might be more evident.

To assess the effect of vimentin on cell surface sensing, here, we use polyacrylamide hydrogels that model the elastic and viscoelastic properties of real tissues. Using wild-type (vim +/+) and vimentin-null (vim −/−) mouse embryonic fibroblasts (mEF), we find that, unlike substrate stiffness, small changes in substrate viscoelasticity have profound effects on how vimentin impacts cell spreading. Unlike elastic substrates, the loss of vimentin significantly reduces cell spread area on substrates with viscous dissipation. Our results suggest vimentin intermediate filaments are a significant contributor to cellular mechano-sensing and could drive differences in cell spreading and motility in tissue.

## Materials and Methods

### Cell Culture

Wild-type mEFs and vimentin-null mEFs were kindly provided by J. Ericsson (Abo Akademi University, Turku, Finland) and maintained in Dulbecco’s Modified Eagle’s Medium (DMEM) + 4.5 g/L glucose, l-glutamine, and sodium pyruvate (Corning). The culture medium was supplemented with 10% fetal bovine serum (Hyclone), 1% penicillin streptomycin (Fisher Scientific) with 25 mM HEPES (Fisher Scientific), and 1% nonessential amino acids (Fisher Scientific). Cell cultures were maintained at 37 °C with 5% CO_2_. Cultures were passaged when they reached 70% confluence.

### Immunofluorescence

Cells were fixed for immunofluorescence using 4% paraformaldehyde (Fisher Scientific). Cell membranes were permeabilized with 0.05% Triton-X (Fisher BioReagents) in PBS for 15 min at room temperature and blocked with 1% bovine serum albumin (BSA) (Fisher BioReagents) for 30 min at room temperature. For vimentin visualization, cells were incubated with primary antivimentin polyclonal chicken antibody (Novus Biologicals) diluted 1:200 in 1% BSA in PBS for 2 h at room temperature; the secondary antibody antichicken Alexa Fluor 488 (Invitrogen) was used at a dilution of 1:1000 in 1% BSA in PBS incubated in the dark for 1 h at room temperature. For visualizing paxillin, we use primary antipaxillin mouse antibody (BD Biosciences) diluted 1:400 in 1% BSA in PBS and incubated for 2 h at room temperature; secondary antibodies were antimouse Alexa Fluor 633 or antimouse Alexa Flour 647 (Invitrogen) at a dilution of 1:1000 in 1% BSA in PBS incubated in the dark for 1 h at room temperature. Cells were stained using Hoechst 33342 (Molecular Probes) at a concentration of 1:1000 in 1% BSA in PBS to detect DNA; cells were also stained using rhodamine phalloidin 565 (Invitrogen) at a dilution of 1:200 in 1% BSA in PBS to detect actin. Cells were incubated for 1 h at room temperature after staining for both DNA and actin.

Cells were imaged using a Leica DMi8 (Leica) equipped with a spinning disk X-light V2 Confocal Unit using a HC PL APO 40×/1.19 W CORR CS2 water immersion objective or using a Nikon Ti-E microscope with Perfect Focus (Nikon Instruments) equipped with a Yokogawa CSU-W1 spining disk confocal unit (Yokogawa), Andor Zyla CMOS camera (Andor Technologies), and 60× Oil Immersion Objective with a 1.49 NA (Nikon Instruments). Images were acquired using the VisiView software (Visitron Systems) or Nikon Elements Software (Nikon Instruments) and analyzed with ImageJ (NIH).

### Bright Field Imaging and Cell Area Measurement

Bright field imaging was performed using a Nikon Eclipse Ti (Nikon Instruments) inverted microscope equipped with an Andor Technologies iXon em+ EMCCD camera (Andor Technologies). Cells were maintained at 37 °C and 5% CO_2_ using a Tokai Hit (Tokai-Hit) stage top incubator and imaged using a Pan Fluor (NA of 0.3) 10× objective. Cell areas were traced manually using ImageJ software. A minimum of at least 60 cells were analyzed over 3+ independent experiments per condition. Box-and-whisker plots and a chart of the cell area data are displayed in Figure S1.

### Focal Adhesion and Stress Fiber Analysis

Fluorescent images of paxillin and F-actin staining were analyzed to determine if a cell contained distinct focal adhesion complexes or stress fibers, respectively. Images were analyzed in ImageJ. We quantified a cell as containing stress fibers if the cell contained an F-actin bundle at least 5 μm in length, which are large enough to be resolved yet small enough to capture stress fibers that only span part of the cell area. When scoring paxillin, we ignored the inner cell cytoplasmic paxillin and chose to focus on the paxillin localized to the edges of the cell area. We scored a cell as containing focal adhesion complexes if a bright spot could be identified on the outer edge of the cell when looking at the paxillin stain. Focal adhesions on glass substrates were counted manually.

### Linear Polyacrylamide Synthesis

Gels are prepared as described previously.^16^ A solution of 0.02% acrylamide in distilled water was degassed until no more bubbling was visible and the solution was cold to touch. Linear PAA polymerization is initiated with the addition of 0.024% ammonium per sulfate (APS; Fisher Scientific) and 0.050% tetramethylethylendiamine (TEMED; Fisher Scientific). The solution is set at room temperature to polymerize for 2 h to allow full polymerization of polyacrylamide, yielding a viscous fluid composed of long linear chains of polyacrylamide.

### Hydrogel Synthesis

Elastic gels are synthesized by making a solution of 8% acrylamide and 0.15% bis-acrylamide (Fisher Scientific); the remaining volume is distilled water. Polymerization is initiated by the addition of 0.25% TEMED and 0.0075% APS. Both types of gel solutions were transferred to a glass coverslip and allowed to polymerize for 20 to 40 min until set. After the gel has polymerized, the gel is stored for up to 1 week in a well tray filled with PBS until the experiment. Concentrations of elastic and viscoelastic gel components are listed in Table 1.

**Table 1:** Recipes for Elastic and Viscoelastic Gels^a^

component	elastic gel (%)	viscoelastic gel (%)
acrylamide	8	8
bis-acrylamide	0.15	0.15
linear PAA	0	2.9
TEMED	0.25	0.25
APS	0.0075	0.0075

a For gels treated with NHS, 10% of distilled water was substituted for 0.04% NHS.

To facilitate cell adhesion with the substrate, collagen I (Corning) is covalently bonded to either the elastic component of the network or both the elastic and viscoelastic components of the network with NHS (Fisher Scientific) or Sulfo-SANPAH (Fisher Scientific), respectively. To bond collagen to only the elastic part of the network, 0.04% of the polymerizing solution is replaced with NHS. To covalently bond collagen to both the elastic and the viscoelastic components of the gel, the surface of the gels is covered by a 0.01% solution of Sulfo-SANPAH in distilled water. The Sulfo-SANPAH is then activated by exposure to ultraviolet light, and gels are washed for three times with PBS. After chemical activation, gels were incubated in a 50 μg/mL collagen I solution in 50 mM HEPES (Fisher Scientific) with pH = 8.2 for 2 h at room temperature. Gels were washed three times with PBS and sterilized with an ultraviolet lamp.

### Rheological Measurement

Rheology measurements were performed on a Malvern Panalytical Kinexus Ultra+ rheometer (Malvern Panalytical) using a 20 mm diameter parallel plate geometry. The elastic and viscoelastic gel solutions are polymerized at room temperature between the rheometer plates at a gap height of 1 mm. The time evolution of polymerization is monitored by applying a small oscillatory shear strain of 2% at a frequency of 1 rad/s for 30 min. The values for the elastic shear modulus and viscous shear modulus of each gel are determined by their plateau value once the gel has fully polymerized. Stress relaxation measurements were performed using a fully polymerized sample by applying a constant shear strain of 5% and tracking the resulting stress relation with time.

### Statistical Analysis

Data is presented as a mean value ± standard error (SE). Each experiment was performed at least twice. A two-way ANOVA with a posthoc Tukey’s test was used to determine statistical significance unless otherwise noted. The Fisher exact test was used to determine statistical significance for the proportion of cells exhibiting actin stress fibers and paxillin patches. Denotations: *, *p* ≤ 0.05; **, *p* ≤ 0.01; ***, *p* < 0.001; n.s., *p* > 0.05.

## Results

### Formulation of Elastic and Viscoelastic Polyacrylamide Hydrogels for Cell Culture

**Figure 1:**
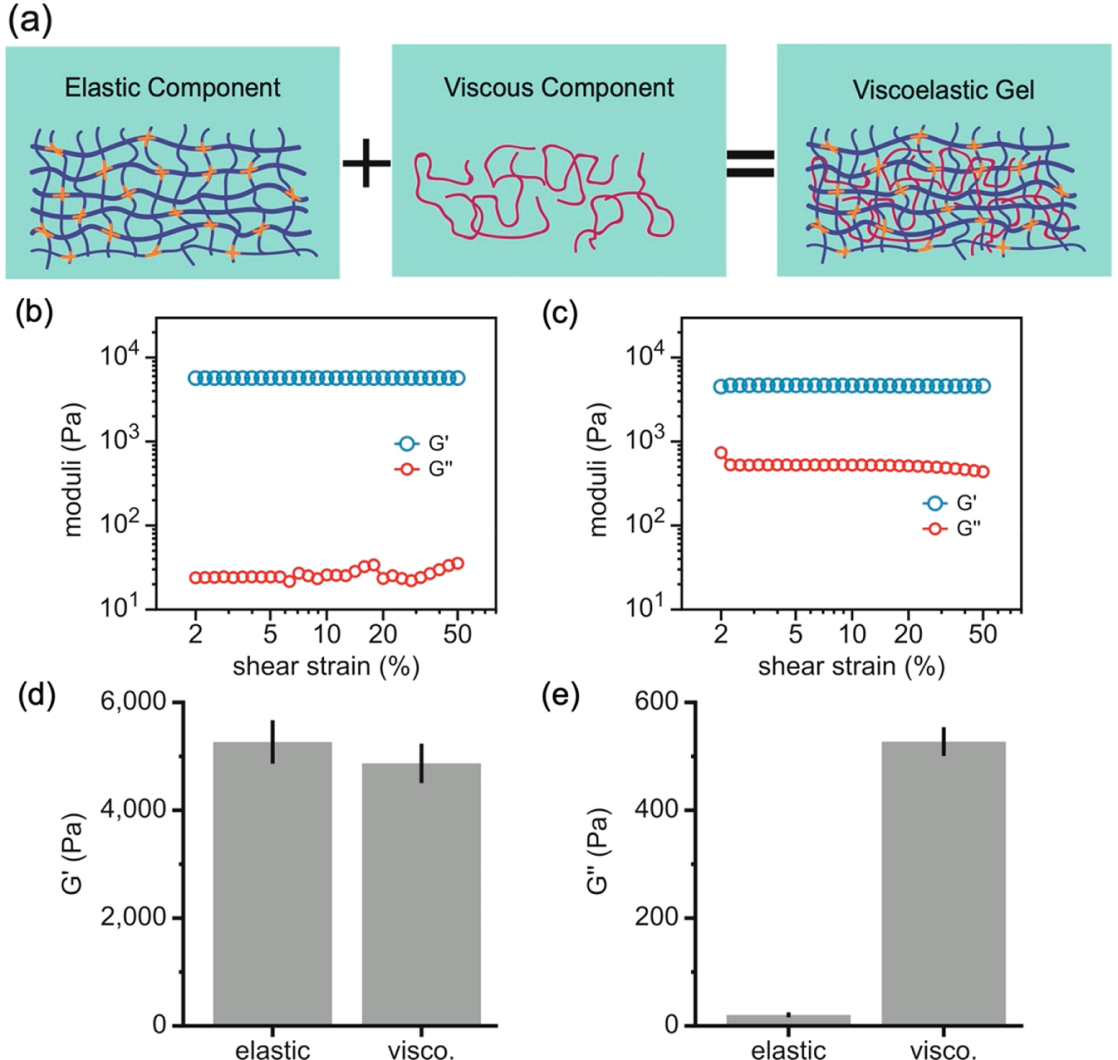
Characterization of viscoelastic polyacrylamide gels. (a) Schematic illustrating the elastic and viscous components of the viscoelastic gel. Acrylamide, cross-linker, and linear PAA chains are shown in blue, orange, and pink, respectively. Representative shear strain sweep measurements of elastic (b) and viscoelastic (c) hydrogels. The storage modulus *G*′ is approximately the same between the two gel types and is constant over a large range of shear strain values. The loss modulus *G*″ is much less than the viscoelastic case and is also much less in comparison to the shear storage modulus (5000 Pa) as expected in the elastic gel, while *G*″ is finite and constant over varying shear strain in the viscoelastic gel. In (d) and (e), the average elastic shear modulus (*G*′) and viscous shear modulus (*G*″) at 2% strain are shown for both elastic and viscoelastic gels, respectively. *N* = 3+ gels per condition; error bars denote standard error.

To examine the effects of substrate viscous dissipation on cell spreading, we prepared polyacrylamide (PAA) hydrogels with elastic and viscoelastic material properties. Elastic PAA gels are a common model system for soft cell culture substrates. Once polymerized, acrylamide and bis-acrylamide form a linearly elastic network with time-independent responses to stress. To form a viscoelastic hydrogel, a dissipative element of linear PAA chains is incorporated into the network. Linear PAA is synthesized by polymerizing acrylamide with TEMED and APS in absence of the cross-linker bis-acrylamide for 2 h to allow full polymerization, yielding a viscous solution of long linear chains of fully polymerized PAA.^16^ When preparing viscoelastic PAA gels, 2.9% of the water is replaced by linear PAA, and when the gel is polymerized, the linear chains become enmeshed inside of the elastic network of the gel but are not cross-linked into the elastic part of the network, allowing viscous dissipation due to flow of the linear chains. Viscoelastic gel polymerization was done in a similar process to elastic polyacrylamide gels. A schematic of the viscoelastic gels is shown in Figure 1a, where the linear PAA chains (pink) are integrated into the elastic cross-linked PAA network (blue and orange). The dissipative component of these gels relaxes in a time dependent response to an applied stress, imparting viscoelastic behavior to the gel.

The mechanical properties of the gels are characterized by a shear storage elastic modulus *G*′ and a viscous loss modulus *G*″ via oscillatory rheology. To determine the effects of viscous dissipation independently of substrate stiffness on cell spreading, the gels were designed with a fixed storage modulus (*G*′ = 5 kPa) but variable loss modulus *G*″ for the elastic (*G*″ = 0 Pa) and viscoelastic (*G*″ = 500 Pa) gels as measured at a frequency of 1 rad/s and 2% shear strain amplitude (Figure 1). The loss modulus of the viscoelastic gel is thus 10% of its elastic moduli, which is in the 10–20% range of real tissue.^16^

If the time scale of substrate relaxation is similar to the time scale of cellular mechanosensing, the substrate relaxation may provide important feedback for cells attempting to spread on viscoelastic substrates. To determine the time dependence of force dissipation on viscoelastic substrates, we performed stress relaxation measurements (Figure S2). A shear strain of 5% is continuously applied to the gel, and the resulting stress relaxation is measured with time (*t*). The stress relaxes to a finite value above zero, indicating the viscoelastic gels behave as a viscoelastic solid. The decrease in stress can be captured by an exponential decay function of the form , where σ is shear stress, *t* is time, τ is the time constant of stress relaxation, and the variables *A*, *B*, and *C* are fitting parameters. By fitting this relationship to the data, we obtain a characteristic time scale (τ = 4.1 ± 0.4 s), which is expected to be relevant to cellular motion in the extracellular matrix environment.^31^

**Figure 2:**
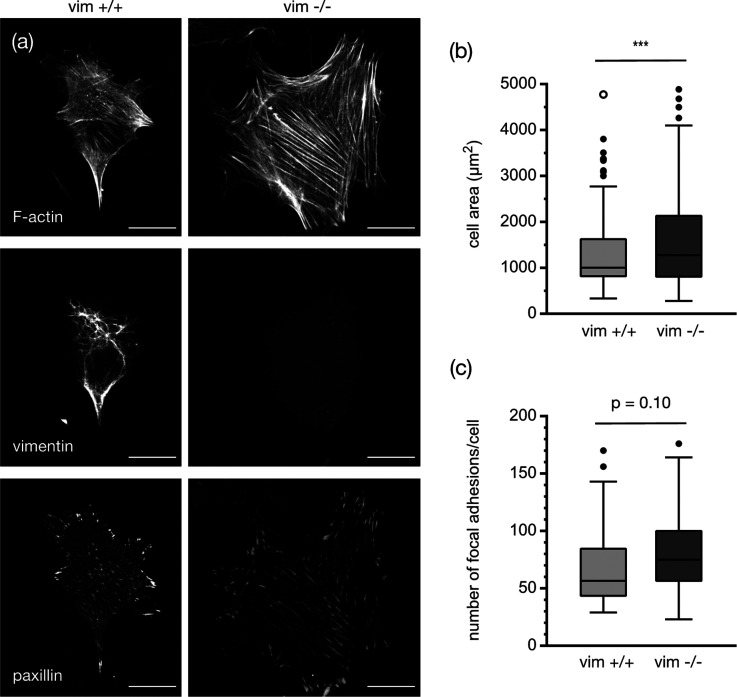
Representative images of both wild-type (vim +/+) and vimentin-null (vim −/−) mouse embryonic fibroblasts (mEFs) on glass slides. (a) Confocal images showing actin, vimentin, and paxillin. Scale bar, 20 μm. (b) Spread area of wild-type and vimentin-null cells on collagen-coated glass coverslips. *N* = 3 independent trials per experimental condition; *n* ≥ 100 cells per condition. (c) Quantification of focal adhesions per cell from paxillin staining. *N* = 3 independent trials; *n* ≥ 40 cells.

To determine how cells spread on viscoelastic substrates and their dependence on intermediate filaments, we conducted our studies with mouse embryonic fibroblasts (mEFs) derived from wild-type (vim +/+) and vimentin-null (vim −/−) mice (Figure 2). Figure 2 shows immunofluorescence images of these two cell lines cultured on traditional glass coverslips. Glass and tissue culture plastic are common substrates for cell growth though they present cells with a rigidity much greater than those found in the *in vivo* tissue environment.^1^ On glass, mEFs adopt highly spread-out morphologies with abundant stress fibers and focal adhesions. Despite the absence of a major cytoskeletal protein, the vim −/– mEFs have similar mean spread areas compared to the vim +/+ mEFs with approximately 1300 ± 50 μm^2^ for vim +/+ mEF and 1800 ± 100 μm^2^ for vim −/– mEF. The stress fibers also appear similar if not more abundant in the vim −/– mEF, consistent with prior studies,^25,28,32^ and might be related to a recent report that suggests vimentin sequesters actin stress fiber formation through RhoA and its guanine nucleotide exchange factor GEF-H1.^33^ Similarly, vim −/– cells have just as many paxillin patches (indicative of focal adhesion) as wild-type cells, consistent with prior reports.^27,34^

### Vimentin Mediates Cell Spreading on Viscoelastic Substrates

**Figure 3:**
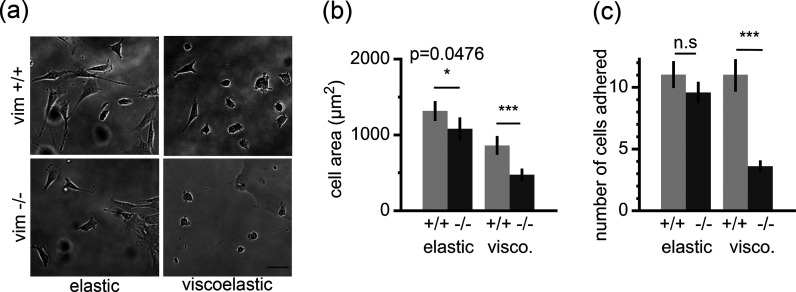
Vimentin enhances cell spreading on viscoelastic substrates. (a) Bright field images of wild-type mouse embryonic fibroblasts (vim +/+) and vimentin-null mouse embryonic fibroblasts (vim −/−) after 24 h of spreading on elastic and viscoelastic gels coated with 50 μg/mL collagen I on the elastic component of the matrix. Scale bar = 30 μm. (b) Average projected cell area of mEFs after 24 h (*N* = 3+ independent trials per condition, 60+ total cells per condition). (c) Average number of cells attached on the gel after 24 h. Cells are counted inside of a 0.5 mm^2^ imaging window (20+ total images analyzed per experimental condition). Statistical significance was determined using a two-way ANOVA with a posthoc Tukey test.

To determine the effects of vimentin on cell spreading on soft and viscoelastic substrates, we cultured wild-type and vimentin-null mouse embryonic fibroblasts (mEFs) (Figure 3) on elastic and viscoelastic polyacrylamide gels (Figure 3a). To facilitate cell attachment to the substrates, the hydrogels were covalently linked with either collagen I bound to the elastic component of the hydrogels or both the elastic and viscous components of the viscoelastic hydrogels (Materials and Methods). The presence of linear PAA in the viscoelastic gels does not affect the presentation of the adhesive ligand, as shown previously with fluorescently labeled fibronectin and atomic force microscopy measurements.^16^ Both gel types were prepared on top of glass coverslips to facilitate bright field and fluorescence microscopy. Figure 3 shows bright field images of cells 24 h after plating on elastic and viscoelastic substrates with collagen I bound to the elastic component of the matrix (Figure 3b). On purely elastic 5 kPa gels, the loss of vimentin did appear to slightly reduce cell spread area (Figure 3b) from 1316 to 1081 μm^2^. This is approximately a 20% reduction with a 0.476 *p*-value that is very close to the common statistical significance threshold (*p* = 0.05).

Both vim +/+ and vim −/– mEFs were less spread on the viscoelastic substrates compared to the elastic substrates, but the effect was much stronger for cells lacking vimentin (Figure 3b, Table S1). We found that wild-type cells reduce their cell spread area by approximately 33% from 1316 μm^2^ on elastic substrates to 860 μm^2^ on viscoelastic substrates. In comparison, vimentin-null cells reduced their cell spread by 45% from 1081 to 475 μm^2^. Perhaps most evident is the relative change in cell area between the two cell types on viscoelastic substrates. On the viscoelastic gels, the null cells had a mean cell spread area 45% smaller than wild-type cells (*p* < 0.001) compared to the 20% reduction on the 5 kPa substrates. While gels with varying amounts of viscous dissipation can be formed, as detailed in refs (35 and 36), here, we have chosen to focus on two conditions, elastic (*G*′ = 5 kPa, *G*″ = 0 kPa) and viscoelastic (*G*′ = 5 kPa, *G*″ = 0.5 kPa), because they present the clearest difference in cell spread area. We compared cell areas on gels with *G*″ = 200 and 500 Pa and found cell spread areas with no statistical difference (data not shown), consistent with prior reports.^16^

The effect of viscoelasticity on cell spreading was most evident when collagen I was presented on the elastic component on the substrate. When collagen I was presented on both the elastic and viscous components, there was not a significant difference in cell spread area for either cell type used here (Figure S3). This was consistent with prior measurements of mEF on these substrates,^16^ and the reason is not entirely clear. One hypothesis is that the populations of cell–matrix contacts transducing a viscoelastic signal differ from those transducing elastic signals, and the coexistence of these two signals act in such a way to promote cell spreading. Here, we choose to focus on the case where collagen I is only coated on the elastic component, so the cells only interact with the cross-linked matrix in which the vimentin’s effects are most evident.

From the time lapse movies, cells can be seen moving and spreading on the viscoelastic substrates. Many of the balled-up cells can be seen forming protrusions and attempting to spread but were not successful. This was seen particularly in the vimentin-null case where cell extensions formed and attempted to reach out; however, they often failed, and the cell remained in a balled-up state anchored at the same position for many frames (staying in the same location for hours) but did not spread. In some cases, the cells clearly detach from the substrate or fail to adhere altogether and presumably die in the suspension.^37^ These observations suggest that the round cells are still alive and adhered to the surface but unable to spread effectively on the viscoelastic substrates.

Vimentin also facilitated cell attachment on soft viscoelastic substrates. To quantify cell–substrate adhesions, we counted the total number of adherent cells after 24 h (Figure 3). On both elastic and viscoelastic substrates, wild-type cells were more successful in remaining adhered to the substrate when compared to vimentin-null cells. This observation was more striking on viscoelastic substrates, where it was seen that on average wild-type cells were 3-fold more likely than vimentin-null cells to remain adhered (Figure 3c). Our data on wild-type mEFs are largely consistent with prior work on 3T3 cells.^16^ In particular, there is a decrease in cell spread area on viscoelastic gels compared to elastic ones, and this change in spread area is associated with fewer stress fibers and focal adhesions yet does not correspond to a decrease the number of wild-type cells adhered to the viscoelastic gels. While many of the vimentin-null cells are poorly spread and rounded up, time lapse videos show that these cells often extend and retract protrusions and remain in the same place on the gel but do not further spread, suggesting a weak adherence with the substrate, whereas wild-type cells spread more efficiently (SI Videos 1 and 2). In some cases, the cells clearly detach from the substrate or fail to adhere altogether, leading to decreased numbers of cells on the substrate.

### Effects of Substrate Viscoelasticity on Cell–Substrate and Cell–Cell Interactions

**Figure 4:**
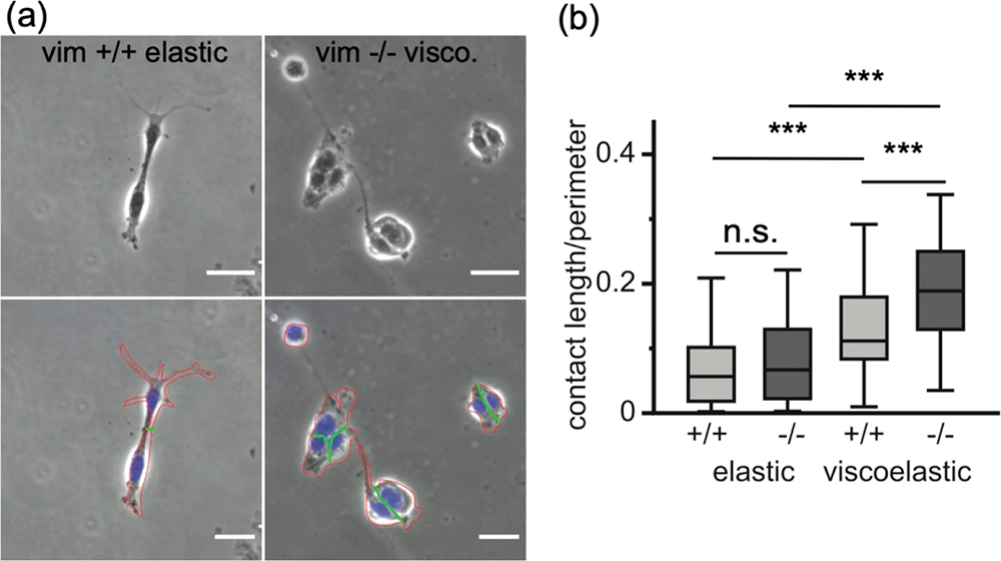
Cell–cell contacts facilitate cell attachment to the substrates with viscous dissipation. (a) Wild-type and vimentin-null cells on viscoelastic substrates. Scale bar = 30 μm. Cell nuclei Hoechst staining is overlaid in blue. Cell perimeters are traced in red, and cell–cell contact lengths are traced in green. (b) Quantification of cell–cell contacts. In vimentin-null cells, cells form greater contact with neighboring cells, as measured by the ratio of the cell contact length to the perimeter of neighboring cells. Data obtained from *N* ≥ 3 individual experiments per condition; total numbers of pairs of cells analyzed per experimental conditions ≥ 90 cells. Error bars denote standard error.

Cell spreading depends on the mechanical properties of the substrate matrix but also direct contact-mediated cell–cell interactions. We observed different rates of cell clustering behavior between our wild-type and vimentin-null mEFs on viscoelastic substrates. Vimentin-null mEFs adhered to viscoelastic substrates were noticeably more likely to be in pairs or a small cluster of cells rather than spread on their own, whereas cells with vimentin or on elastic substrates did not cluster tightly (Figure 4). Cell–cell interactions regulate many cell signal processes and cellular functions. This is consistent with literature results, which show that cell–cell interactions regulate vimentin-dependent collagen contraction.^23^ Because the total number of cell–cell connections or clusters is a function of both the cell spread area and the number of cells adhered, which varies under each condition, we quantified cell–cell interactions by another metric, the length of direct contact between two touching cells compared to the total perimeter of the two cells. Here, the increase of the contact length/perimeter indicates increasing cell–cell contact over cell–substrate contact. Figure 4a shows two examples of these cell–cell interactions. For wild-type cells on elastic substrates, cell–cell contacts are small compared to the cell’s contact with the substrate, whereas the vimentin-null cells on viscoelastic substrates are strongly coupled. To normalize the cell–cell contact with cell spread area, we define the cell–cell contact by dividing the contact length by the total perimeter of the pair of cells. As shown in Figure 4b, we find that substrate viscoelasticity increases cell–cell contact for both cell types, indicating that cell–cell interactions promote adhesion on viscoelastic substrates. While cell–cell contact is similar between wild-type and vimentin-null meFs on elastic substrates, cell–cell contact increases more than 150% for vimentin-null cells compared to wild-type cells on the viscoelastic substrates. Taken together, these results indicate that vimentin may facilitate adhesion to viscoelastic substrates by promoting cell–matrix adhesions over cell–cell interactions.

### Substrate Viscoelasticity Mediates Cytoskeletal and Focal Adhesion Organization

**Figure 5:**
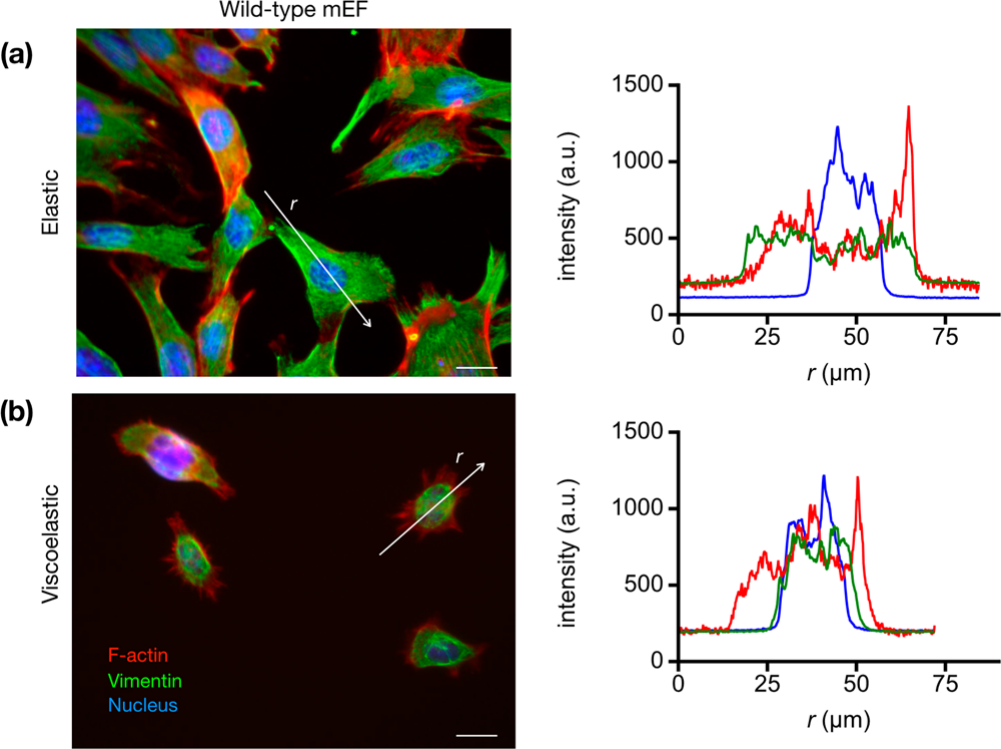
Viscoelastic substrates decrease cell spreading and alter vimentin organization. Representative immunofluorescence images of wild-type cells on (a) elastic and (b) viscoelastic gels. On elastic gels, the vimentin cytoskeletal network extends out toward the periphery of the cell, while on viscoelastic gels, the vimentin network is more collapsed around the nucleus. This is highlighted by the line scans through the immunofluorescence images (on right). Vimentin, green; actin, red; DNA, blue. Scale bar, 20 μm.

Next, we analyzed the organization of vimentin networks on elastic and viscoelastic substrates. Vimentin networks are apparent in wild-type mEFs on all substrates, but the organization of the vimentin networks varied, as shown in immunofluorescence images in Figure 5. On the elastic substrates, vimentin was spread throughout the cytoplasm of the cells with filamentous bundles extending toward the periphery of the cells. Filamentous vimentin bundles such as these have been implicated in load-bearing units in the cell, contributing to proper alignment of actin-based cell traction stress.^38^ However, on viscoelastic substrates, the vimentin network was more condensed in a mesh-like cage around the cell nucleus and vimentin filamentous strands were less evident, which is likely due in part because the cells are less spread^39,40^ (Figure 3). The localization of vimentin around the cell nucleus on the viscoelastic substrates is evident by line scans through the immunofluorescence images of the cells (Figure 5).

**Figure 6:**
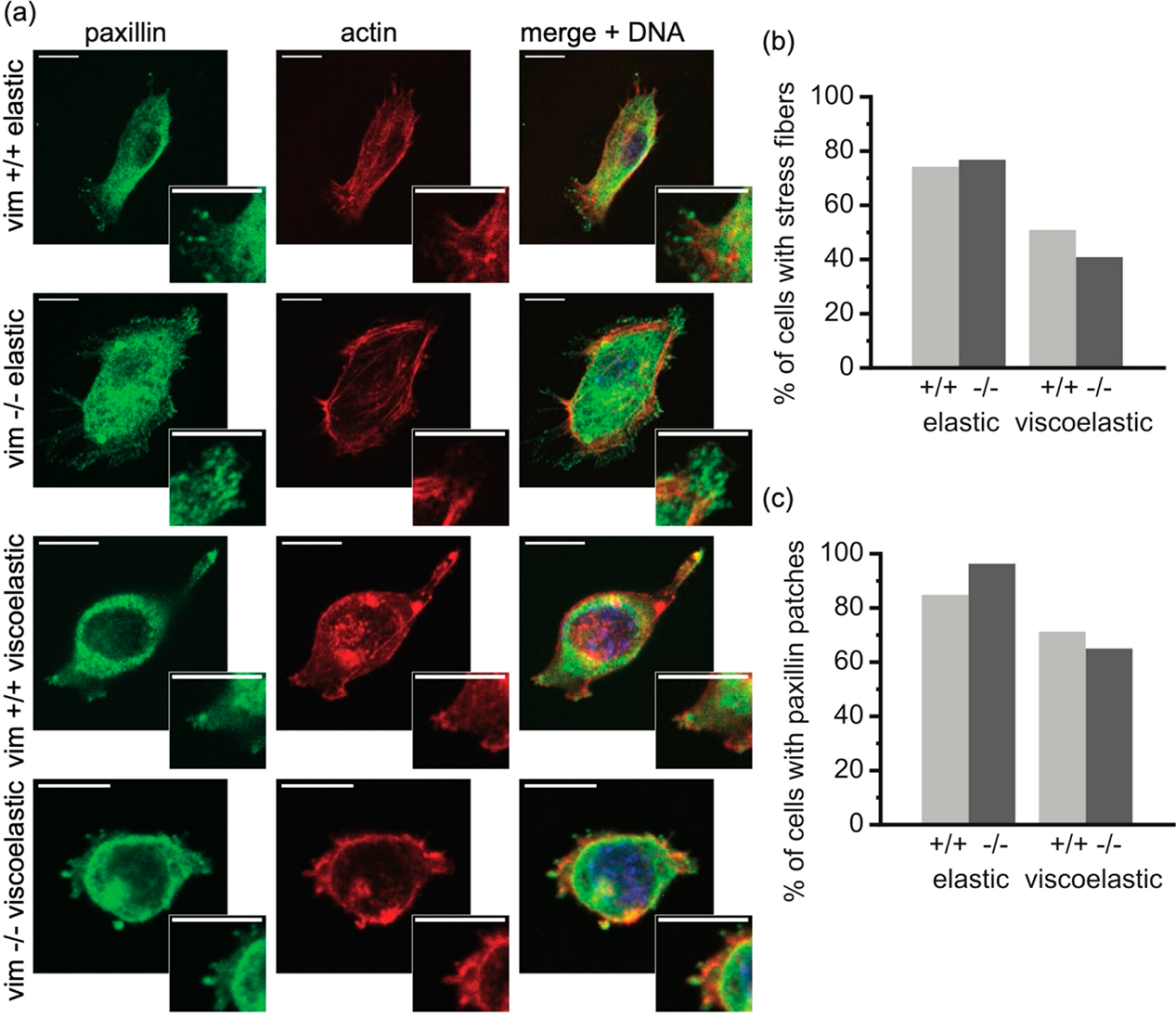
Distribution of actin stress fibers and paxillin focal adhesion patches on elastic and viscoelastic substrates. (a) Immunofluorescence images of actin (red), paxillin (green), and DNA (blue) on elastic and viscoelastic substrates. On the left are spinning disk confocal images of both wild-type (vim +/+) and vimentin-null (vim −/−) mouse embryonic fibroblasts that have been incubated for 24 h on elastic and viscoelastic substrates coated with collagen I. Scale bar, 10 μm. The right presents the percentage of cells that exhibit actin stress fibers (b) and paxillin patches (c). Data collected from *N* ≥ 3 independent experiments per condition with 60+ cells per condition. Statistical significance was calculated with the Fisher’s exact test.

The effects of vimentin on cell spreading on viscoelastic substrates (Figure 3) suggest force-bearing adhesions and stress fibers would be affected by vimentin. To adhere and spread on a substrate, cells form focal adhesion complexes that link cell integrins to the substrate. Paxillin forms a major part of the focal adhesions, and paxillin clusters are indicative of focal adhesion formation. Paxillin patches and actin fibers were visualized on elastic and viscoelastic substrates using immunofluorescence and confocal microscopy (Figure 6). On 5 kPa elastic gels, wild-type and null cells developed much smaller focal adhesion patches compared to those on glass substrates (Figure 2). Most cells displayed paxillin patches (>80%) and actin stress fibers (>70%) on elastic substrates. On viscoelastic substrates, however, there were fewer cells displaying paxillin patches (60%) and stress fibers (40%). The number of cells with focal adhesions and stress fibers were found to be similar between wild-type and vimentin-null cells for both elastic and viscoelastic conditions. This result on 5 kPa elastic gels is interesting given that on glass substrates null cells display more adhesions.^27^ For viscoelastic substrates, the similar presence of adhesions and stress fibers is surprising because the loss of vimentin reduces cell spreading (Figure 3) and less spread cells typically display fewer adhesions and stress fibers. Taken together, our results indicate that vimentin impacts focal adhesion formation differently on soft versus rigid substrates, and spreading on viscoelastic substrates may be less dependent on the presence of paxillin patches at least as measured by immunofluorescence.

## Discussion and Conclusion

Cell spreading is a complex process that probes the fundamental interactions between the behavior of the cell and its coupling to the underlying substrate matrix. This process depends on the cellular cytoskeleton and focal adhesion complexes that attach the cell to the substrate. While the role of actin and microtubules in the mechanosensitive response of the cell have been studied in great detail,^41−43^ much less is known regarding the role of intermediate filaments. The actomyosin network in particular serves as the main force-generating component of the cell and is thus central to differences in cell spreading on substrates with varying stiffnesses. Recent work using latrunculin A inhibitors show actin is also necessary for spreading on substrates with viscous dissipation.^44^ Here, we demonstrate that vimentin is also an important element of cellular mechanical sensing, particularly on substrates with viscous dissipation.

We found that wild-type (vim +/+) and vimentin-null (vim −/−) mEFs spread similarly on soft elastic substrates (*G*′ = 5 kPa), in agreement with prior experiments,^19^ but cells lacking vimentin have significantly less spreading on viscoelastic substrates. On viscoelastic gels, wild-type cells are less spread and have fewer paxillin patches, indicating a weaker cell–substrate interaction. The loss of vimentin is correlated with a fewer number of substrate adherent cells and stronger cell–cell interactions (Figure 4).

The strong effect of substrate viscoelasticity on vimentin-dependent cell spreading might be surprising given that vimentin is largely dispensable for cell spreading on elastic substrates.^23^ One possible reason for the high sensitivity to vimentin expression for cells spreading on viscoelastic substrates may be the mechanical stability provided by the vimentin networks. Filamentous vimentin networks are less dynamic^45^ and more viscoelastic^19^ compared to the actin and microtubule networks in the cytoskeleton. Thus, the stabilizing effect of vimentin on microtubule orientation^46^ and actin-based stresses^33,38^ may be particularly evident for cell spreading on viscoelastic substrates, which act to dissipate and effectively lessen cell-generated traction stresses.

Another hypothesis is that the effect of vimentin in regulating cell adhesion could promote cell spreading on viscoelastic substrates. Cells are thought to sense their substrate through a “motor-clutch” mechanism.^5^ In this context, focal adhesions act as molecular clutches, physically linking the cellular cytoskeleton to the extracellular matrix substrate. These physical linkages transmit traction forces to the underlying substrates. Motor clutches are generally assumed to act as slip bonds with a dissociation rate that increases with the amount of force it bears by coupling the cell with the substrate. As motors bind with the substrate, they generate friction and resist the retrograde flow of F-actin filaments. This allows actin polymerization to push the leading edge of the cell forward, which ultimately results in cell spreading. Recent motor-clutch models have been developed to capture the effects of adhesion dynamics and substrate viscoelasticity on cell spreading.^2,44^ On viscoelastic substrates, the dynamics of focal adhesion assembly and disassembly compete with substrate viscous dissipation. On viscoelastic substrates, cells feel a time-dependent effective stiffness that decreases over a characteristic substrate relaxation time scale. This results in weaker adhesions and faster retrograde flows, which decrease cell spreading. Prior experiments using Fluorescence Recovery After Photobleaching (FRAP) analysis of GFP-paxillin showed that the rate of paxillin turnover in MCF-7 cells is significantly higher in cells with high levels of vimentin expression.^29^ If vimentin increases the binding time of the clutches, then clutches can quickly bind and break without forming large stable focal adhesions. This is consistent with recent measurements on rigid glass substrates^34^ showing that vimentin-null cells have more but smaller integrin clusters and paxillin focal adhesion patches. In this case, the binding time scales are larger than the lifetime of a focal adhesion, resulting in an effective “frictional slippage” regime that stalls retrograde flow and promotes cell spreading. Since focal adhesion turnover is slower in cells lacking vimentin, they are predicted to sense a lower effective substrate stiffness. In this case, the clutch binding time is less than the lifetime of the motor clutches, resulting in a “load and fail” regime. Adhesive forces are expected to be smaller than the frictional slippage regime in vimentin expressing cells, resulting in increased retrograde flow and reduced cell spreading. Thus, vimentin might promote cell spreading on viscoelastic substrates by increasing focal adhesion dynamics. This effect could work in parallel with vimentin’s stabilizing effects on the cell cytoskeleton to promote and aid spreading on viscoelastic substrates.

Taken together, our results indicate a new function of vimentin intermediate filaments in modulating cellular responses to viscoelastic environments that has important implications for understanding cell and tissue functions. Intermediate filaments play diverse roles in a range of cell and tissue functions^47,48^ and are important to maintaining cell morphology and adhesion.^27^ Vimentin IF in particular has been implicated in cataracts,^49^ coronaviruses,^50−52^ wound healing,^53^ and many forms of metastatic cancer.^54−56^ Our results indicate that vimentin promotes cellular adhesion and motility through viscoelastic environments, such as extracellular matrices and *in vivo* tissue. More broadly, our results show that vimentin contributes to how cells respond to viscoelastic properties of the extracellular matrix where applied stresses dissipate on time scales relevant to cellular mechanical sensing.

## Supporting Information Available

Wild-type mouse embryonic fibroblast on NHS viscoelastic gel (AVI)
Vimentin-null mouse embryonic fibroblast on NHS viscoelastic gel (AVI)
Additional experimental details, materials, and methods (PDF)

## Supplementary Material

mt1c01046_si_001.avi

mt1c01046_si_002.avi
