# Supplementary material for: Vimentin Intermediate Filaments Mediate Cell Morphology on Viscoelastic Substrates
Source: ACS Appl Bio Mater. 2022 Jan 7;5(2):552–61. doi: 10.1021/acsabm.1c01046 (PMC8864613; doi:10.1021/acsabm.1c01046)
Supplement: Supplementary file 3 — mt1c01046_si_003.pdf [file mt1c01046_si_003.pdf]

Supplementary Material for

Vimentin intermediate filaments mediate cell morphology on viscoelastic substrates

Maxx Swoger[†,‡], Sarthak Gupta[†,‡], Elisabeth E. Charrier[¶], Michael Bates[§], Heidi Hehnly[§], Alison E. Patteson[†,‡,*]

[†]Physics Department, Syracuse University, NY, 13244, USA

[‡]BioInspired Institute, Syracuse University, NY, 13244, USA

[¶]Institute of Medicine and Engineering, University of Pennsylvania, PA, 13210, USA

[§]Biology Department, Syracuse University, NY, 13244, USA

[*]E-mail:aepattes@syr.edu

Supplementary Figures & Tables

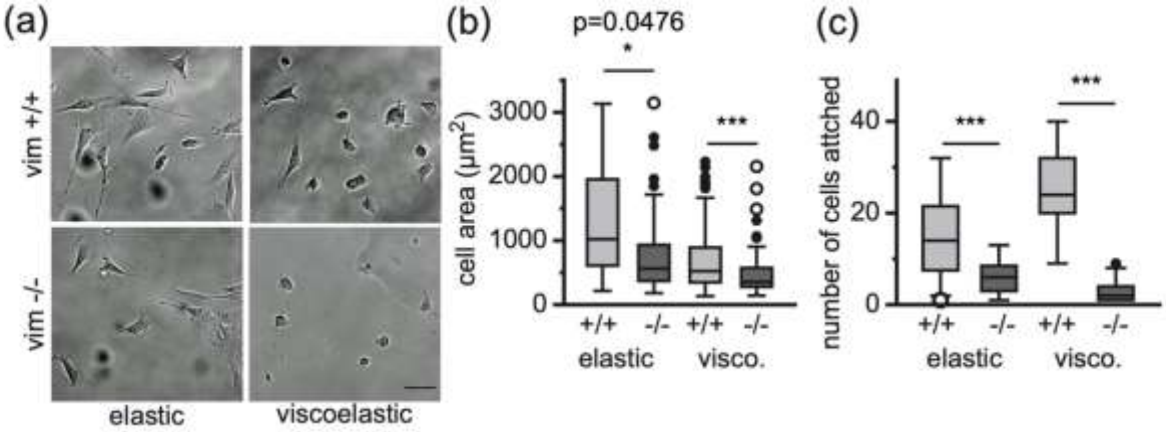

SI. Fig. 1. Vimentin enhances cell spreading on viscoelastic substrates. (a) Bright field images of wild-type mouse embryonic fibroblasts (vim +/+) and vimentin-null mouse embryonic fibroblasts (vim -/-) after 24 hr of spreading on elastic and viscoelastic gels, coated with 50 μg/mL collagen I on the elastic component of the matrix. Scale bar = 30 μm. (b) Average projected cell area of mEFs after 24 hr (N = 3+ independent trials per condition, 60+ total cells per condition). (c) Average number of cells attached on gel after 24 hours. Cells are counted inside of a 0.5 mm^2 imaging window (20+ total images analyzed per experimental condition). Statistical significance was determined using a two-way ANOVA with a post-hoc Tukey test.

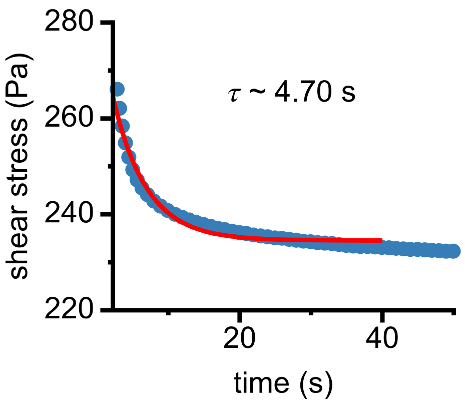

SI. Fig. 2. Characteristic stress-relaxation data from four independent experiments. Shear stress is shown in blue dots as a function of time for four separate stress relaxation experiments (a-d). Data is fitted to an arbitrary exponential function of the form $\sigma(t) = Aexp(-{}^{(t-B)}/_{\tau}) + C$, where σ is shear stress, t is time, τ is the time constant of stress relaxation, and the variables A, B, and C are fitting parameters. The time constant for each fit is presented within the panel of the plot and the average value for this time constant is τ =4.1 ± 0.4. The R^2 value of the fit is 0.98. (N=4 independent samples for stress relaxation data).

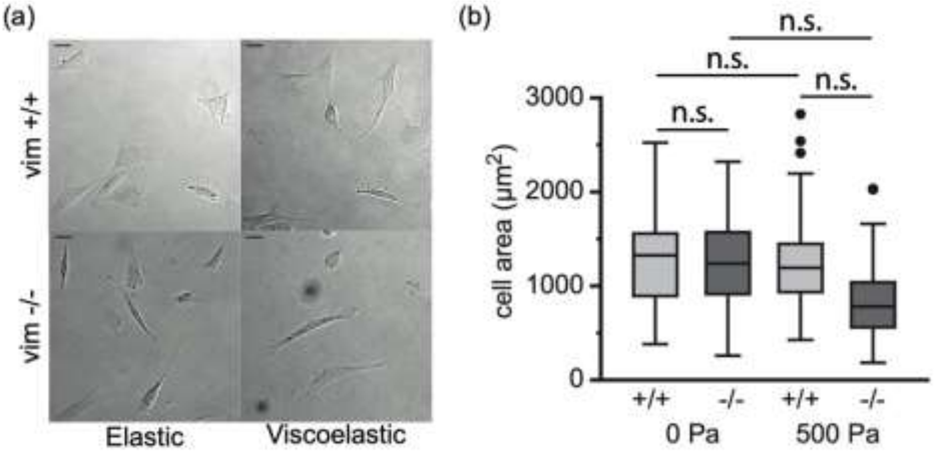

SI. Fig. 3. Vimentin does not enhance cell spreading on viscoelastic substrates when both the elastic and viscoelastic components of the gels are coated with collagen. (a) bright field images of wild-type mouse embryonic fibroblasts (vim +/+) and vimentin-null mouse embryonic fibroblasts after spreading for 24 hours on gels coated with Sulfo-SANPAH, allowing collagen to bond to both the elastic and viscous component of viscoelastic gels. Gels are coated with 50 μg/mL collagen I, scale bar = 30 μm. (b) average cell area of wild-type and vimentin-null cells after 24 hours. (N=2 individual experiment with n ≥ 60 cells measure in each experiment. Error bars denote standard error. Statistical significance was determined using a two-way ANOVA with a post-hoc Tukey test.

	Cell type	Mean Area (µm^2)	Standard error (µm^2)	N
Glass	Wild-type	1265	47	221
Glass	Null	1754	110	243
Elastic	Wild-type	1316	111	126
Elastic	Null	1081	132	97
Viscoelastic	Wild-type	860	106	92
Viscoelastic	Null	475	62	93

SI Table 1. Cell spread data on glass, elastic (G′ = 5 kPa, G′′ = 0 Pa), and viscoelastic (G′ = 5 kPa, G′′= 500 Pa) gels. Surfaces are coated with 50 µg/mL rat tail collagen I.

Supplementary Video Captions

Supplementary Video 1: Wild-type mouse embryonic fibroblast on NHS viscoelastic gel (G'=5 kPa; G''=500 Pa). Video taken at 10x over first 4 hours of spreading.

Supplementary Video 2: Vimentin-null mouse embryonic fibroblast on NHS viscoelastic gel (G'=5 kPa; G'' = 500 Pa). Video taken at 10x over first 4 hours of spreading.
